# Preparation and isolation of isobenzofuran

**DOI:** 10.3762/bjoc.13.263

**Published:** 2017-12-12

**Authors:** Morten K Peters, Rainer Herges

**Affiliations:** 1 Otto-Diels-Institut für Organische Chemie, Christian-Albrechts-Universität, Otto-Hahn-Platz 4, 24118 Kiel, Germany

**Keywords:** [4 + 2] cycloaddition, Diels–Alder, isobenzofuran, trapping reagent

## Abstract

The synthesis, isolation and characterization of isobenzofuran are described in this publication. Isobenzofuran is of general interest in synthetic and physical organic chemistry because it is one of the most reactive dienes known. A number of synthetic pathways have been published which all suffer from disadvantages such as low yields and difficult purification. We present a synthetic pathway to prepare isobenzofuran in laboratory scale with high yields, from affordable, commercially available starting materials.

## Introduction

**Scheme 1:**
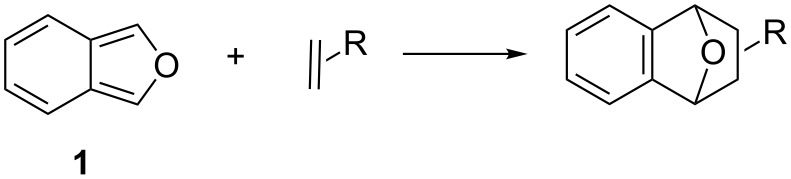
Diels–Alder reaction of isobenzofuran and formation of a benzene ring in the cycloadduct.

Isobenzofurans have been described as the most reactive dienes for Diels–Alder reactions [1–5]. Their high reactivity is mainly due to the resonance energy gained by formation of a benzene ring in the cycloaddition product (Scheme 1) [6].

Isobenzofurans have been extensively used as 4 electron (diene) components in Diels–Alder reactions, and moreover in other cycloaddition reactions such as [4 + 3], [4 + 4], [8 + 8] and [4 + 6] additions [7–10]. Highly strained alkenes and alkynes have been trapped with isobenzofurans. 1,3-Diphenylisobenzofuran is the preferred trapping reagent for singlet oxygen and is used to quantify the generation of ^1^O_2_ in photodynamic therapy [4,11]. The most important synthetic application is probably the preparation of annulated polycyclic aromatic hydrocarbons by cycloaddition to arynes [8,12]. However, the high reactivity of isobenzofurans comes at the cost of low stability [13]. 1,3-Diphenylisobenzofuran is reasonably stable and commercially available and therefore the most frequently used isobenzofuran derivative. The parent system isobenzofuran (IBF, **1**) is about 10 times more reactive but generally described as a reagent that is difficult to prepare and to purify [1]. Therefore, it should be generated in situ, and used without isolation.

## Results and Discussion

We now present a reliable and convenient synthesis providing high yields of isobenzofuran. In contrast to previous reports, the compound is stable for more than 8 months in pure form as a solid at −15 °C. The half-life of IBF (**1**) in solution (150 mM, 27 °C, toluene-*d*_8_) is about 12 h. A half-life of 2 h in CDCl_3_ has been previously reported [14]. Isobenzofurans are light sensitive. Warrener et al. reported on [8 + 8] cycloaddition products upon irradiation. Depending on the solvent further dimers are formed [14].

Several procedures have been published for the synthesis of IBF (**1**). The key step in the majority of the reported methods is a retro Diels–Alder reaction [6,13,15–17]. Fieser and Haddadin [17] describe IBF as a transient intermediate and Warrener and Wege [13,15] isolated IBF at −80 °C on a cold finger. The disadvantages of these methods are high reaction temperatures during vacuum pyrolysis and multistep syntheses of the precursors.

The alternative way to synthesize IBF (**1**) is 1,4-elimination of 1,3-dihydro-1-methoxyisobenzofuran (**7**, DMIBF), which provides access to IBF (**1**) at ambient temperature [18]. Three methods have been published to prepare **7** (DMIBF). Reduction of phthalic acid (**2**) or phthalic acid ester **3** to 1,2-bis(hydroxymethyl)benzene (**4**) [19], acid promoted ring closure, and subsequent oxidation with hypochlorite in the presence of methanol gives **7** in a moderate yield of 65% [18].

Alternatively, phthalide **5** has been reduced to 1,3-dihydroisobenzofuran-1-ol (**6**) and methylated to DMIBF (**7**) [8]. However, yields in our hands are quite low.

**Scheme 2:**
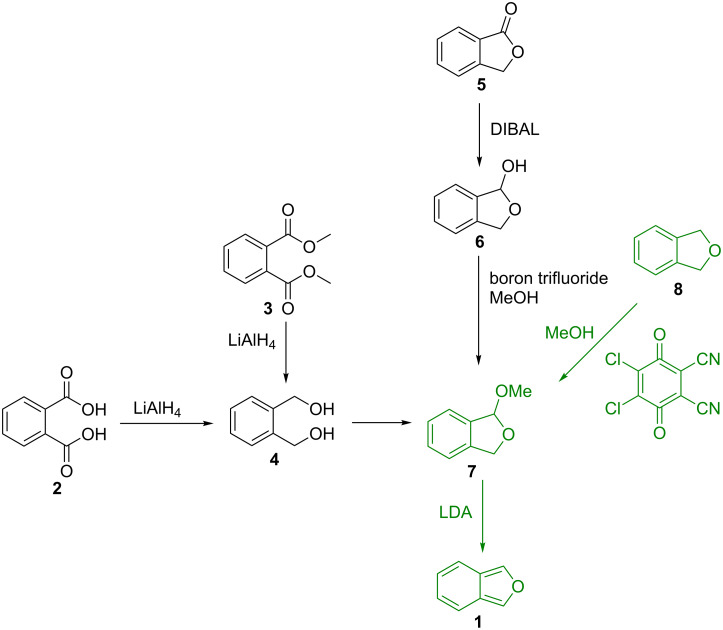
Different approaches for the synthesis of IBF (**1**).

It is known that benzyl ethers are prone to oxidative functionalization [20]. 2,3-Dichloro-5,6-dicyano-1,4-benzoquinone (DDQ) has been used to selectively oxidize benzyl ethers to acetals in the presence of alcohols [21]. Following a procedure of Doyle et al. we reacted commercially available phthalan (**8**) with DDQ and methanol in dry dichloromethane under a nitrogen atmosphere at room temperature, and obtained DMIBF (**7**) with a yield of 85% (Scheme 2) [22]. DMIBF (**7**) was treated with freshly prepared lithium diisopropylamide (LDA) in benzene and IBF (**1**) was obtained as a solution in benzene which was washed with aqueous NH_4_Cl to remove lithium salts and amines (Scheme 2) [8,18].

**Scheme 3:**
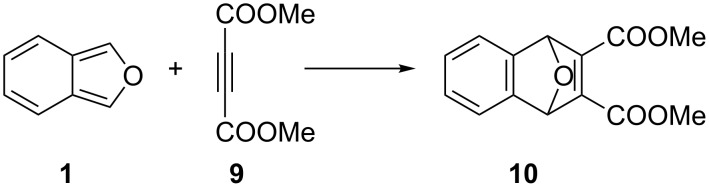
Reaction of in situ prepared IBF (**1**) with DMAD (**9**).

To determine the yield of IBF (**1**), this solution was reacted with acetylenedicarboxylic acid dimethyl ester (DMAD, **9**) and product **10** was obtained with a yield of 78% relative to the precursor DMIBF (**7**, Scheme 3).

To further purify isobenzofuran (**1**), the benzene solution was carefully evaporated and the residue was subjected to column chromatography over silica gel (cyclohexane/ethyl acetate). We were able to isolate IBF (**1**) as a colorless solid (mp 20 °C) with a yield of 66%. The solid compound can be stored for up to 8 months at −15 °C without decomposition (polymerization).

## Conclusion

Isobenzofuran (**1**) is one of the most reactive dienes in Diels–Alder reactions and other cycloadditions. For practical applications it has been generated and reacted in situ, because it rapidly dimerizes or polymerizes in solvents of medium polarity such as chloroform (*t*_1/2_ = 2 h). We observed longer half-lives in low polarity solvents (*t*_1/2_ = 12 h in toluene-*d*_8_, 150 mM, 27 °C). We have been able to purify the compound by chromatography and to isolate it as a colorless solid (mp 20 °C). In crystalline form, it is stable for 8 months at −15 °C without decomposition. Upon oxidative methoxylation of commercially available phthalane (85% yield) [22], and subsequent 1,4-elimination with LDA [18] we obtained isobenzofuran (**1**) in 78% yield (trapping reaction) or in 66% isolated yield after chromatography.

## Experimental

NMR spectra were measured in deuterated solvents (Deutero). Analytic measurements were performed by the following instruments: Bruker CABAV 500neo (^1^H NMR: 500 MHz, ^13^C NMR: 125 MHz) and Bruker AV 600 (^1^H NMR: 600 MHz, ^13^C NMR: 150 MHz). Infrared spectra were recorded on a Perkin-Elmer 1600 Series FTIR spectrometer with an A531-G Golden-Gate-Diamond-ATR-unit. The high-resolution (HR) mass spectra were measured with an APEX 3 FT-ICR with a 7.05 T magnet by co. Bruker Daltonics. Electron impact (EI).

**1,3-Dihydro-1-methoxyisobenzofuran (7)** [22]: 2,3-Dichloro-5,6-dicyano-1,4-benzoquinone (DDQ, 5.00 g, 22.0 mmol), dry dichloromethane (100 mL), methanol (900 μL, 22.2 mmol) and phthalan (**8**, 2.00 g, 16.7 mmol) were dissolved under a nitrogen atmosphere. The reaction mixture was stirred for 13 h at room temperature. The reaction was quenched with aq sodium hydrogen carbonate solution and filtered over Celite. The aqueous phase was extracted three times with dichloromethane. The combined organic layers were dried over magnesium sulfate and the solvent was removed under reduced pressure. The crude product was purified by column chromatography on silica gel (cyclohexane/ethyl acetate 8:2, *R_f_* 0.58) A colorless oil was obtained. Yield: 2.13 g (14.2 mmol, 85%); ^1^H NMR (500 MHz, 300 K, CDCl_3_) δ 7.41 (d, ^3^*J* = 7.3 Hz, 1H), 7.39–7.33 (m, 2H), 7.27 (d, ^3^*J* = 7.4 Hz, 1H), 6.19 (d, ^4^*J* = 2.2 Hz, 1H), 5.22 (dd, ^2^*J* = 12.7 Hz, ^4^*J* = 2.2 Hz, 1H), 5.05 (d, ^2^*J* = 12.7 Hz, 1H), 3.44 (s, 3H) ppm; ^13^C NMR (150 MHz, 300 K, CDCl_3_) δ 140.0, 137.3, 129.3, 127.9, 123.0, 121.0, 107.3, 72.2, 54.2 ppm.

**Isobenzofuran (1, IBF)** [18]: Diisopropylamine (1.43 g, 14.2 mmol) was dissolved in benzene (5.00 mL), and cooled to 0 °C. 2.5 M *n*-butyllithium solution in hexane (6.70 mL) was added dropwise and the mixture was stirred for 15 min. The freshly prepared LDA solution was warmed up to room temperature. Then 1-methoxy-1,3-dihydroisobenzofuran (**7**, 800 mg, 5.33 mmol), dissolved in benzene (8 mL), was added dropwise and stirred for 5 min. The mixture was washed with ammonium chloride solution and then twice with water. The combined organic layers were dried over magnesium sulfate and the solvent was removed under reduced pressure. The crude product was purified by column chromatography on silica gel (cyclohexane/ethyl acetate 8:2, *R_f_* 0.92). The solvent was carefully evaporated at 20 °C. A colorless solid was obtained. Yield: 415 mg (3.52 mmol, 66%); mp 20 °C; IR (ATR): 3138 (w), 3044 (w), 2923 (w), 1774 (w), 1695 (m), 1462 (m), 1428 (m), 1368 (m), 1195 (w), 1043 (s), 976 (s), 950 (s), 888 (s), 871 (m), 758 (s), 672 (m), 635 (s), 601 (s), 539 (s), 496 (s) cm^−1^; ^1^H NMR (600 MHz, 300 K, DMSO-*d*_6_) δ 8.32 (s, 2H), 7.45 (dd, ^3^*J* = 6.8 Hz, ^4^*J* = 2.8 Hz, 2H), 6.86 (dd, ^3^*J* = 6.8 Hz, ^4^*J* = 2.8 Hz, 2H) ppm; ^13^C NMR (150 MHz, 300 K, DMSO-*d*_6_) δ 136.1, 124.2, 123.5, 119.0 ppm; HRMS (EI) *m/z*: [M]^+^ calcd. for C_8_H_6_O, 118.04173; found, 118.04186.

**Dimethyl 1,4-epoxy-1,4-dihydronaphthalene-2,3-dicarboxylate (10):** Dimethyl acetylenedicarboxylate (**9**, 1.00 g, 7.04 mmol) was dissolved in benzene (25 mL) under a nitrogen atmosphere. A freshly prepared solution of isobenzofuran (**1**), which was prepared from DMIBF (**7**, 1.10 mmol, 165 mg), prior to purification by chromatography (see procedure above) was added dropwise and stirred for 16 h at 50 °C. The crude product was purified by column chromatography on silica gel (cyclohexane/ethyl acetate 8:2, *R_f_* 0.27). A colorless oil was obtained. Yield: 223 mg (858 µmol, 78%); IR (ATR): 2953 (w), 1710 (s), 1637 (m), 1435 (m), 1291 (m), 1250 (s), 1211 (s), 1109 (s), 1064 (m), 976 (m), 910 (m), 854 (s), 755 (s), 734 (m), 655 (s) cm^−1^; ^1^H NMR (600 MHz, 300 K, CDCl_3_) δ 7.43 (dd, ^3^*J* = 5.2 Hz, ^4^*J* = 3.0 Hz, 2H), 7.07 (dd, ^3^*J* = 5.2 Hz, ^4^*J* = 3.0 Hz, 2H), 5.96 (s, 2H), 3.80 (s, 6H) ppm; ^13^C NMR (150 MHz, 300 K, CDCl_3_) δ 162.8, 151.2, 146.2, 126.1, 121.4, 84.8, 52.4 ppm; HRMS (EI) *m/z*: [M]^+^ calcd. for C_12_H_12_O_5_, 260.06847; found, 260.06800.

## Supporting Information

File 1 Analytical equipment and methods, experimental procedures and NMR spectra.
