# Supplementary material for: Preparation and isolation of isobenzofuran
Source: Beilstein J Org Chem. 2017 Dec 12;13:2659–62. doi: 10.3762/bjoc.13.263 (PMC5753151; doi:10.3762/bjoc.13.263)

Supporting Information

for

Preparation and isolation of isobenzofuran

Morten K. Peters and Rainer Herges*

Address: Otto-Diels-Institut für Organische Chemie, Christian-Albrechts-Universität, Otto-Hahn-Platz 4, 24118 Kiel, Germany

Email: Rainer Herges* - rherges@oc.uni-kiel.de

* Corresponding author

Analytical equipment and methods, experimental procedures and NMR spectra

Table of Contents

I. Analytical equipment and methods

II. Experimental procedures

III. NMR spectra

I. Analytical equipment and methods

NMR Spectroscopy

NMR spectra were measured in deuterated solvents (Deutero). The degree of deuteration is given in parentheses. ^{1}H NMR spectra in reference to the following signals:

chloroform-*d* (99.8%): δ = 7.26 ppm (s)

DMSO-d_6 (99.8%): δ = 2.50 ppm (quint)

The signal multiplicities are abbreviated as follows:

s: singlet, d: doublet, t: triplet, q: quartet, quint: quintet, m: multiplet, br: broad signal

Measurements were performed by the following instruments:

Bruker CABAV 500neo (^{1}H NMR: 500 MHz, ^{13}C NMR: 125 MHz)

Bruker AV 600 (^{1}H NMR: 600 MHz, ^{13}C NMR: 150 MHz)

IR spectroscopy

Infrared spectra were measured on a Perkin-Elmer 1600 Series FT-IR spectrometer with an A531-G Golden-Gate-Diamond-ATR-unit. Signals were abbreviated with w, m, s and for weak, medium and strong intensities. Broad signals are additionally labeled with br.

Mass spectrometry

The high resolution (HR) mass spectra were measured with an APEX 3 FT-ICR with a 7.05 T magnet by co. Bruker Daltonics. Electron impact (EI). Electrospray ionization (ESI) mass spectra were measured with a Thermo Scientific Q EXACTIVE.

Chromatography stationary phases

For column chromatography purifications silica gel (Merck, particle size 0.040–0.063 mm) was used. R_f values were determined by thin layer chromatography onPolygram® Sil G/UV_{254} (Macherey-Nagel, 0.2 mm particle size).

Elemental analysis

The amount of carbon, hydrogen and nitrogen in a compound was determined with a CHNSO-Elemental analyser Euro EA 3000 Series by co. Euro Vector.

II. Experimental procedures

II.1 Synthesis of phthalic acid ester 3

Phthalic acid (**2**, 6.00 g, 36.2 mol) was dissolved in methanol (130 mL). After that conc. sulfuric acid (6.80 mL) was slowly added dropwise. The reaction mixture was stirred under reflux for 24 h. After cooling down to room temperature the solution was concentrated and added to a mixture of 200 mL dichloromethane/water (1:1). The aqueous phase was extracted three times with dichloromethane. The combined organic layers were dried over magnesium sulfate and the solvent was removed under reduced pressure. A yellow oil was obtained.

Yield.: 6.75 g (34.8 mmol, 96%, Lit. 70%[1])

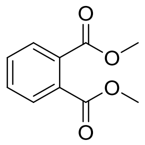

^{1}H-NMR (500 MHz, 300 K, $CDCl_3$): δ = 7.72 (dd, ^3J = 5.7 Hz, ^4J = 3.3 Hz, 2H), 7.53 (dd, ^3J = 5.7 Hz, ^4J = 3.3 Hz, 2H), 3.90 (s, 6H) ppm.

^{13}C-NMR (150 MHz, 300 K, $CDCl_3$): δ = 168.0, 131.9, 131.1, 128.9, 52.6 ppm.

II.2 Synthesis of 1,2-bis(hydroxymethyl)benzene (4)

II.2.1 Method 1:

To a slurry of lithium aluminium hydride (2.50 g, 65.9 mmol, 2.5 equiv) in 315 mL of THF was slowly added a solution phthalic acid (**2**, 4.38 g, 26.4 mol, 1 equiv) in 215 mL of THF at 0 °C under nitrogen. The solution was stirred for 16 h. Thereafter the suspension was filtered and the filtrate was extracted three times with diethyl ether. The combined organic layers were washed with water and dried over magnesium sulfate and the solvent was removed under reduced pressure. A colorless solid was obtained.

Yield.: 940 mg (6.81 mmol, 26%, Lit. 84%[2])

[1] Nakamura, R.; Obora, Y. I.; Ishii, Y. *Advanced Synthesis and Catalysis* **2009,** *351,* 1677–1684.

II.2.2 Method 2:

To a slurry of lithium aluminium hydride (1.95 g, 51.5 mmol, 2.5 equiv) in 315 mL of THF was slowly added a solution phthalic acid ester **3** (4.00 g, 20.6 mol, 1 equiv) in 215 mL of THF at 0 °C under nitrogen. The solution was stirred for 36 h. The solution was filtered and the filtrate extracted three times with diethyl ether. The combined organic layers were washed with water and dried over magnesium sulfate and the solvent was removed under reduced pressure. A colorless solid was obtained.

Yield.: 2.16 g (16.1 mmol, 66%, Lit. 85%[3])

II.2.3 Method 3:

Phthalic acid ester **3** (1.00 g, 5.15 mol) was dissolved in 45 mL of dry THF under nitrogen. A borane tetrahydrofuran complex solution (20.0 mL, 20.0 mmol, 1 M in THF) was added dropwise at 0 °C over 1 h and then stirred for 16 h at room temperature. The reaction mixture was quenched with methanol and the solvent was removed under reduced pressure. A colorless solid was obtained, which was dissolved in dichloromethane and washed twice with water. The solvent was again removed under reduced pressure and a pure colorless solid was obtained.

Yield.: 654 mg (4.74 mmol, 92%)

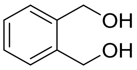

^{1}H-NMR (500 MHz, 300 K, $CDCl_3$): δ = 7.27 (s, 4H), 4.57 (s, 4H), 4.00 (s, 2H, OH) ppm.

^{13}C-NMR (150 MHz, 300 K, $CDCl_3$): δ = 139.3, 129.6, 128.5, 63.8 ppm.

---

[2] Zysman-Colman, E.; Nevins, N.; Eghball, N.; Snyder, J. P.; Harpp, D. N. *J. Am. Chem. Soc.* **2006** , 128, 291 - 304.

[3] D'Ayala, G. G.; Malinconico, M.; Laurienzo, P.; Tardy, A.; Guillaneuf, Y.; Lansalot, M.; D'Agosto, F.; Charleux, B. *J. of Polymer Science* **2014** , 52, 104 - 111.

II.3 Synthesis of 1,3-Dihydro-1-methoxyisobenzofuran 7

II.3.1 Method1:

Diisobutylaluminum hydride (7.11 g, 50.0 mmol in toluene) was added over a period of 45 min to a stirred solution of phthalide (**5**, 6.70 g, 50.0 mmol) in toluene (100 mL) under nitrogen at −78 °C. Ether (50 mL) was added and the solution was stirred for 4 h. The cold bath was removed. Ether (150 mL) was immediately added, followed by saturated sodium chloride solution (100 mL). The layers were separated and the aqueous layer was extracted 4 times with ether 100 mL. The combined organic layers were dried over magnesium sulfate. The solvent was removed under reduced pressure and an oil was obtained, which was then dissolved in methanol (200 mL). Boron trifluoride etherate (1 mL) was added to the solution at 0 °C. After 2 h of stirring, the reaction mixture was poured into saturated sodium chloride solution (450 mL) and extracted 4 times with *n*-pentane (100 mL). The combined organic layers were washed with water and dried over magnesium sulfate and the solvent was removed under reduced pressure. Vacuum distillation gave a colorless liquid.

Yield.: 180 mg (1.20 mmol, 2%, Lit. 69%[4])

II.3.2 Method 2:

A mixture of 1,2-bis(hydroxymethyl)benzene (**4**, 1.50 g, 11.3 mmol), methanol (60 mL) conc. sulfuric acid (2.55 mL) and pentane (120 mL) was stirred while 30 mL of 5.25% aqueous NaOCl was added over a period of 8 h. After 24 h of additional stirring, the phases were separated and the aqueous part was washed twice with pentane (50 mL). The combined organic layers were washed with aqueous bicarbonate and dried over magnesium sulfate. The crude product was purified by column chromatography on silica gel (cyclohexane/ethyl acetate 8:2, R_f 0.58.) A colorless oil was obtained.

Yield.: 1.12 g (7.46 mmol, 66 %, Lit. 65%[5])

[4] Man, Y. M.; Mak, T. C. W.; Wong, H. N. C. *J. Org. Chem.* **1990**, 55, 3214.

[5] Naito, K.; Rickborn, B. *J. Org. Chem.* **1980**, 45, 4061.

II.3.3 Method 3:

see publication

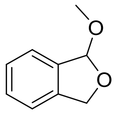

^{1}H-NMR (500 MHz, 300 K, $CDCl_3$): δ = 7.41 (d, ^{3}J = 7.3 Hz, 1H), 7.39-7.33 (m, 2H), 7.27 (d, ^{3}J = 7.4 Hz, 1H), 6.19 (d, ^{4}J = 2.2 Hz, 1H), 5.22 (dd, ^{2}J = 12.7 Hz, ^{4}J = 2.2 Hz, 1H), 5.05 (d, ^{2}J = 12.7 Hz, 1H), 3.44 (s, 3H) ppm.

^{13}C-NMR (150 MHz, 300 K, $CDCl_3$): δ = 140.0, 137.3, 129.3, 127.9, 123.0, 121.0, 107.3, 72.2, 54.2 ppm.

II.4 Synthesis of Isobenzofuran (1)

see publication

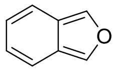

IR (ATR): 3138 (w), 3044 (w), 2923 (w), 1774 (w), 1695 (m), 1462 (m), 1428 (m), 1368 (m), 1195 (w), 1043 (s), 976 (s), 950 (s), 888 (s), 871 (m), 758 (s), 672 (m), 635 (s), 601 (s), 539 (s) , 496 (s) cm^{-1}.

^{1}H-NMR (600 MHz, 300 K, DMSO-d_6): δ = 8.32 (s, 2H), 7.45 (dd, ^{3}J = 6.8 Hz, ^{4}J = 2.8 Hz, 2H), 6.86 (dd, ^{3}J = 6.8 Hz, ^{4}J = 2.8 Hz, 2H) ppm.

^{13}C-NMR (150 MHz, 300 K, DMSO-d_6): δ = 136.1, 124.2, 123.5, 119.0 ppm.

MS: (HR) EI

**calc.**	**found**	**Mass. Diff (ppm)**	**Molecular formula**
**118.04173**	**118.04186**	**-1.2**	**C_8H_6O**

II.5 Synthesis of dimethyl 1,4-epoxy-1,4-dihydronaphthalene-2,3-dicarboxylate (10)

see publication

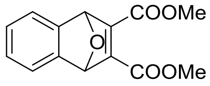

IR (ATR): 2953 (w), 1710 (s), 1637 (m), 1435 (m), 1291 (m), 1250 (s), 1211 (s), 1109 (s), 1064 (m), 976 (m), 910 (m), 854 (s), 755 (s), 734 (m), 655 (s) cm^{-1}.

^{1}H-NMR (600 MHz, 300 K, $CDCl_3$): δ = 7.43 (dd, ^{3}J = 5.2 Hz, ^{4}J = 3.0 Hz, 2H), 7.07 (dd, ^{3}J = 5.2 Hz, ^{4}J = 3.0 Hz, 2H), 5.96 (s, 2H), 3.80 (s, 6H) ppm.

^{13}C-NMR (150 MHz, 300 K, $CDCl_3$): δ = 162.8, 151.2, 146.2, 126.1, 121.4, 84.8, 52.4 ppm.

MS: (HR) EI

**calc.**	**found**	**Mass. Diff (ppm)**	**Molecular formula**
**260.06847**	**260.06800**	**-1.83**	**$C_{12}H_{12}O_5$**

III. NMR spectra

III.1 Phthalic acid ester 3

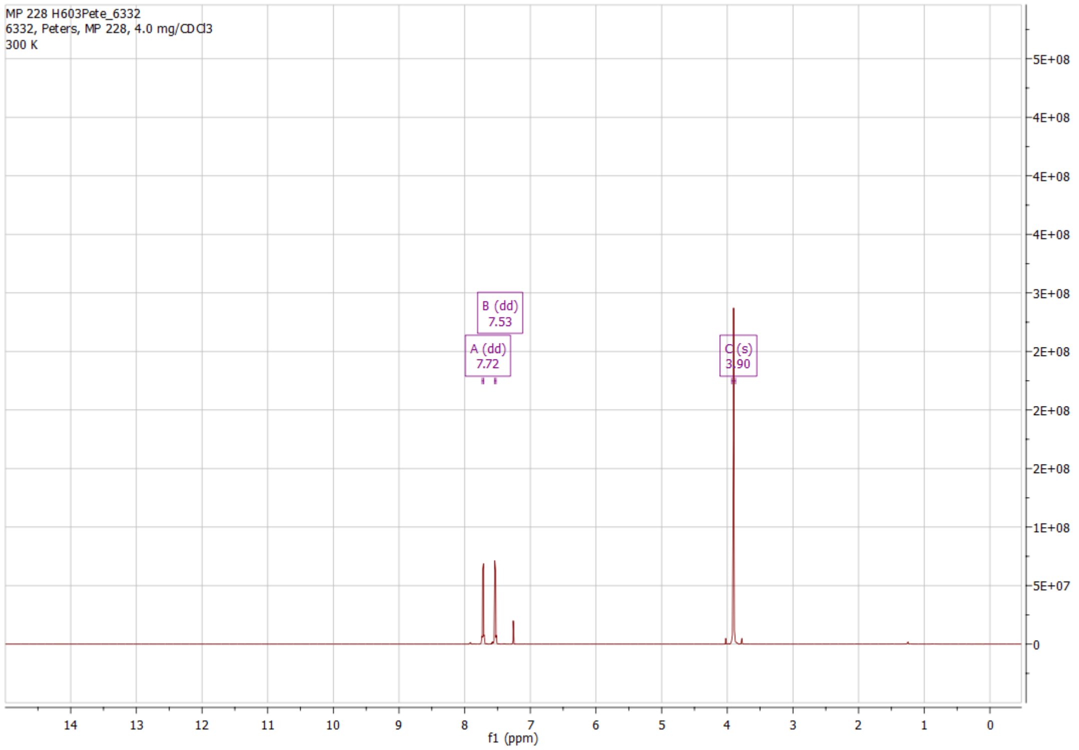

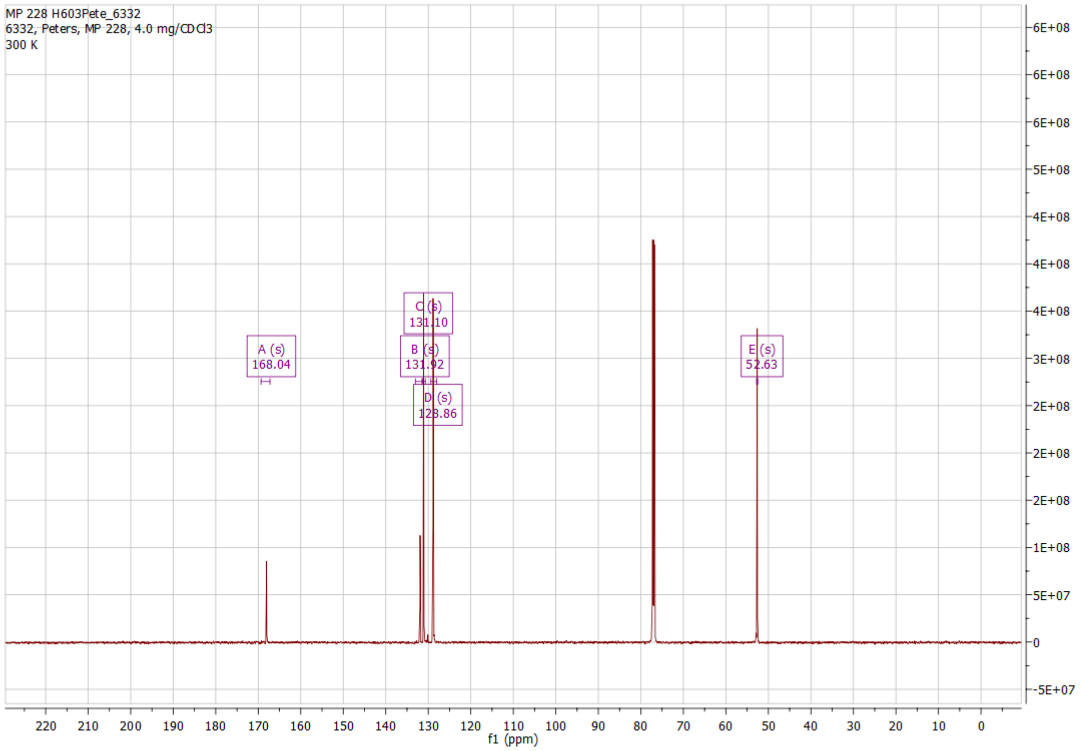

III.2

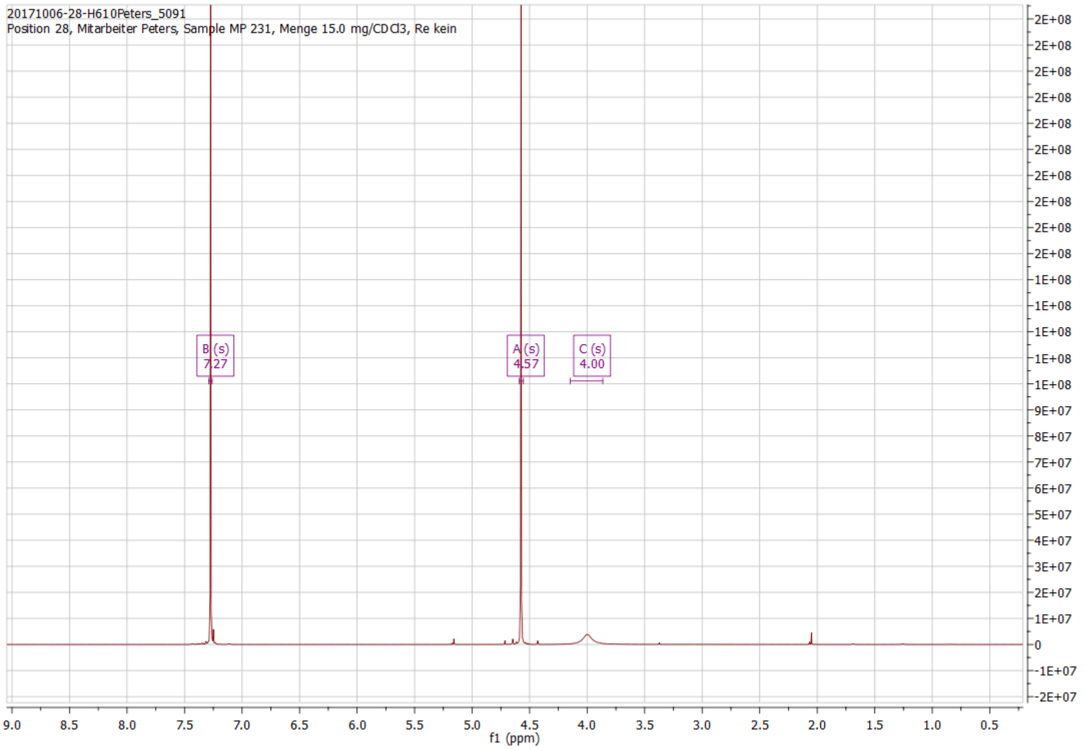
20171006-28-H610Peters_5091
Position 28, Mitarbeiter Peters, Sample MP 231, Menge 15.0 mg/CDCl3, Re kein
B (s)
7.27
A (s)
4.57
C (s)
4.00
f1 (ppm)

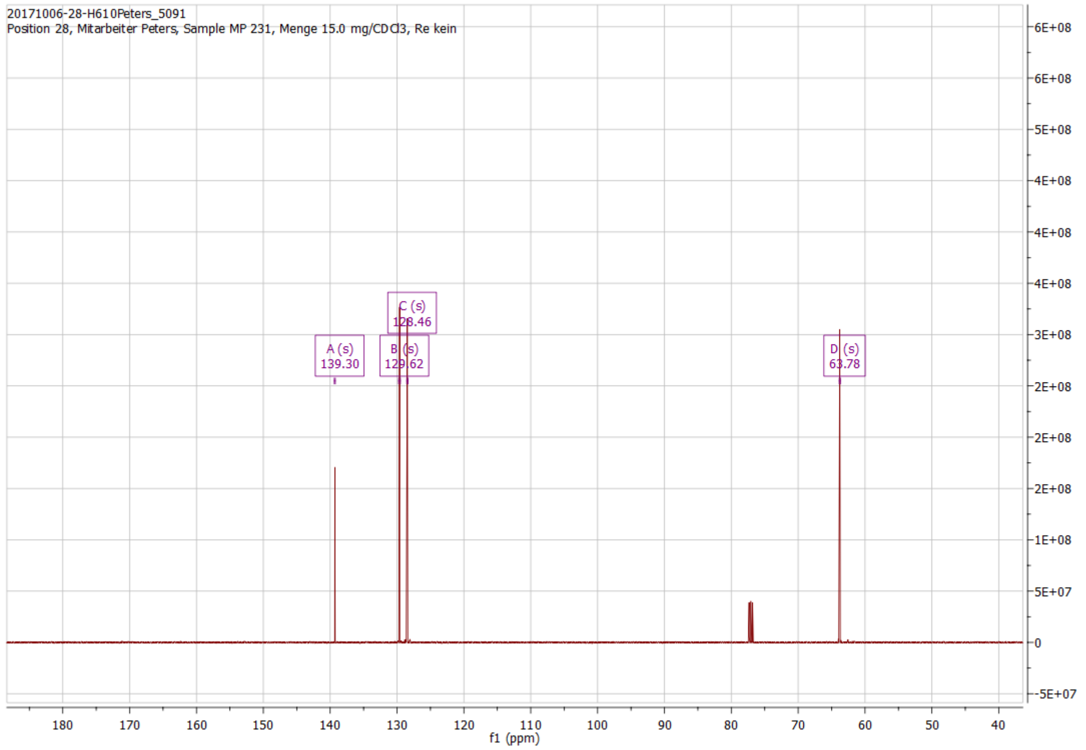
20171006-28-H610Peters_5091
Position 28, Mitarbeiter Peters, Sample MP 231, Menge 15.0 mg/CDCl3, Re kein
C (s)
128.46
A (s)
139.30
B (s)
129.62
D (s)
63.78
f1 (ppm)

III.3 1,3-Dihydro-1-methoxyisobenzofuran (7)

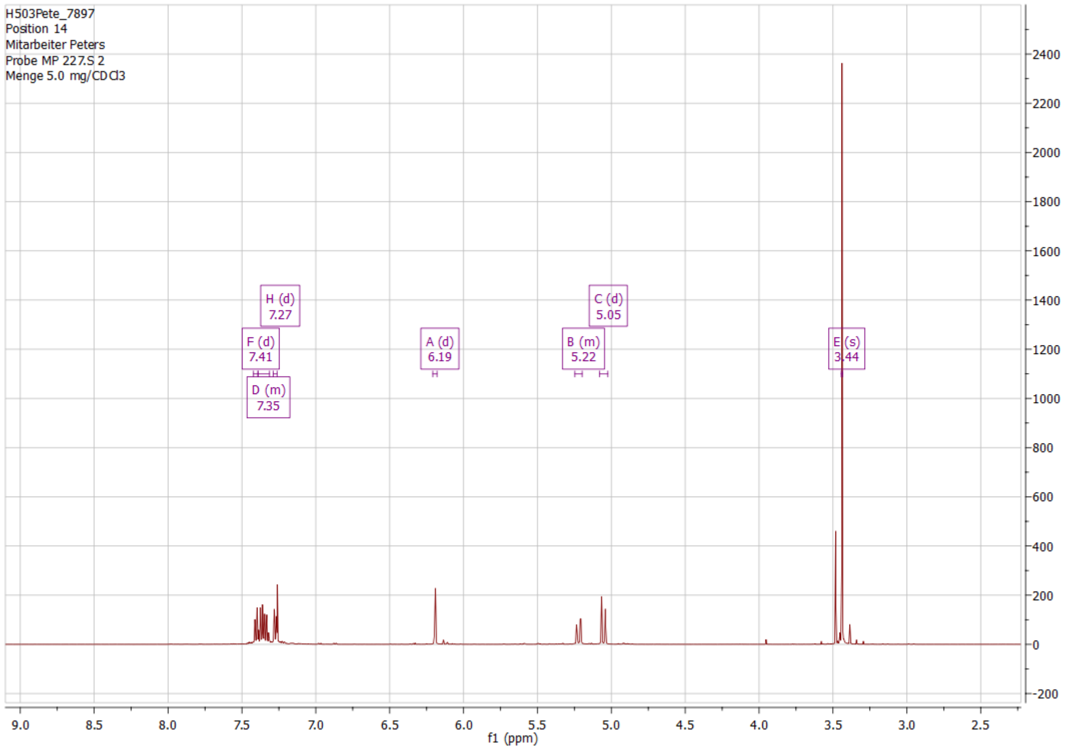

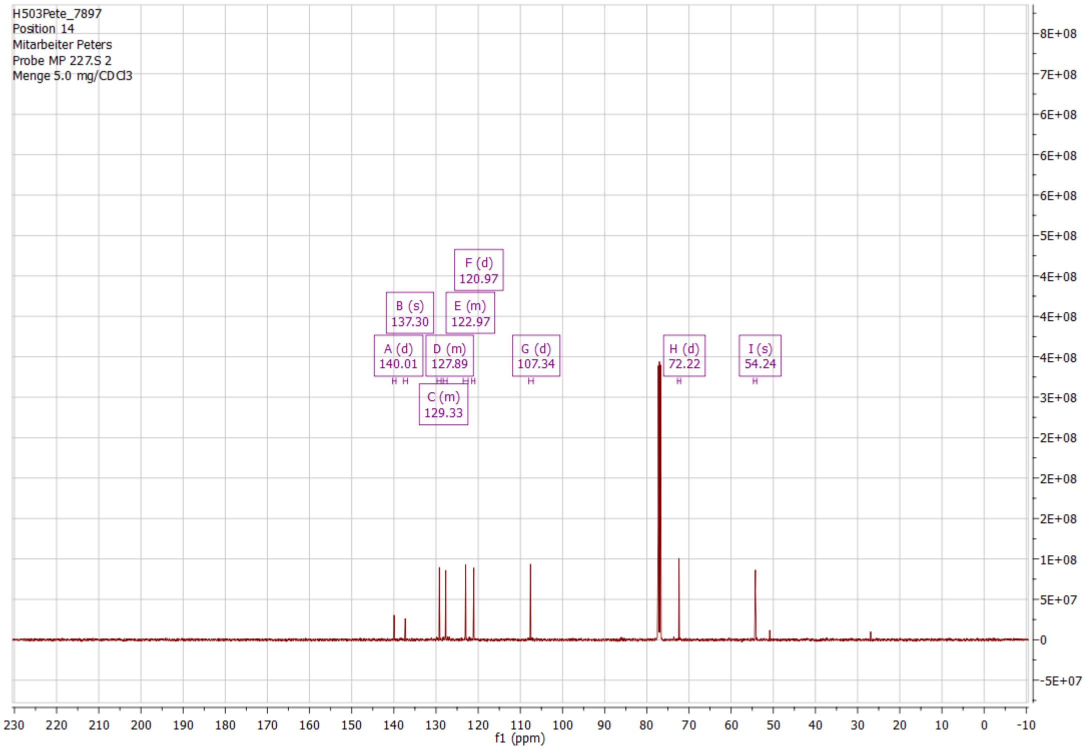

III.4 Isobenzofuran (1)

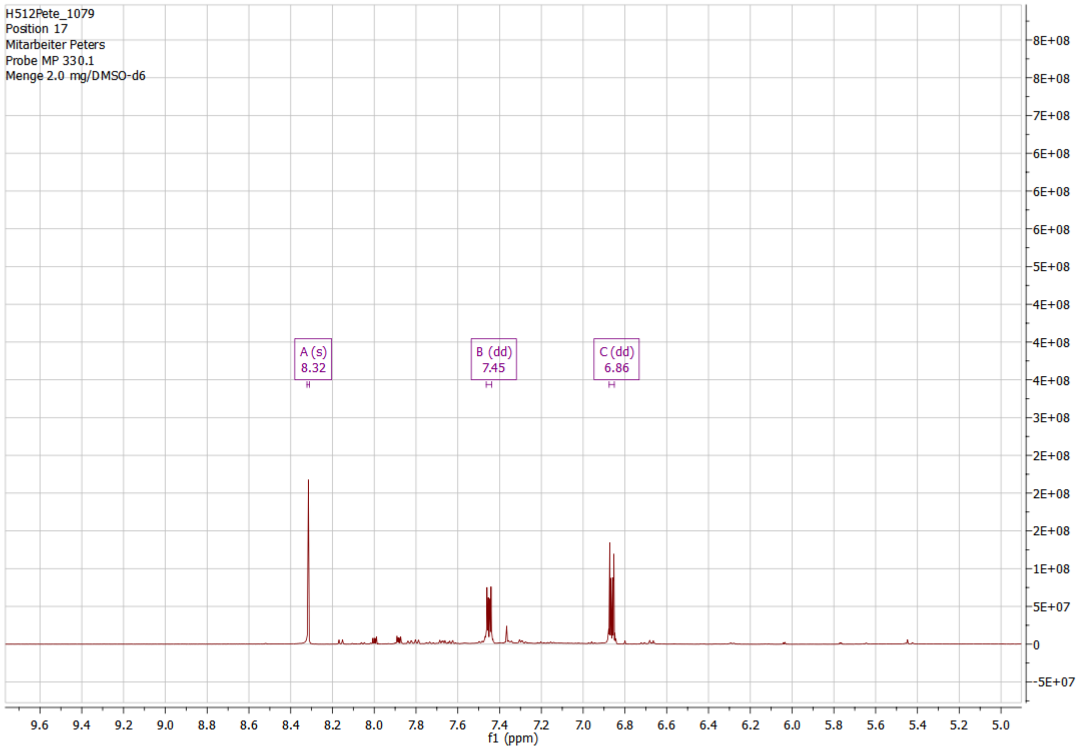

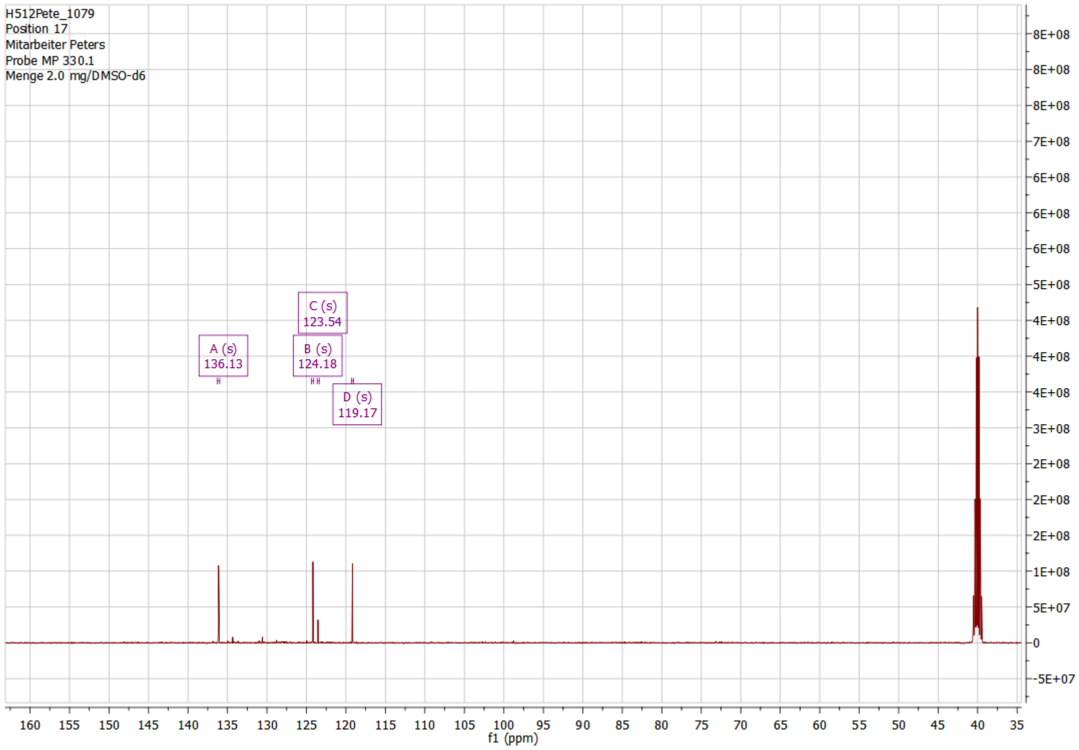

III.5 Dimethyl 1,4-epoxy-1,4-dihydronaphthalene-2,3-dicarboxylate (10)

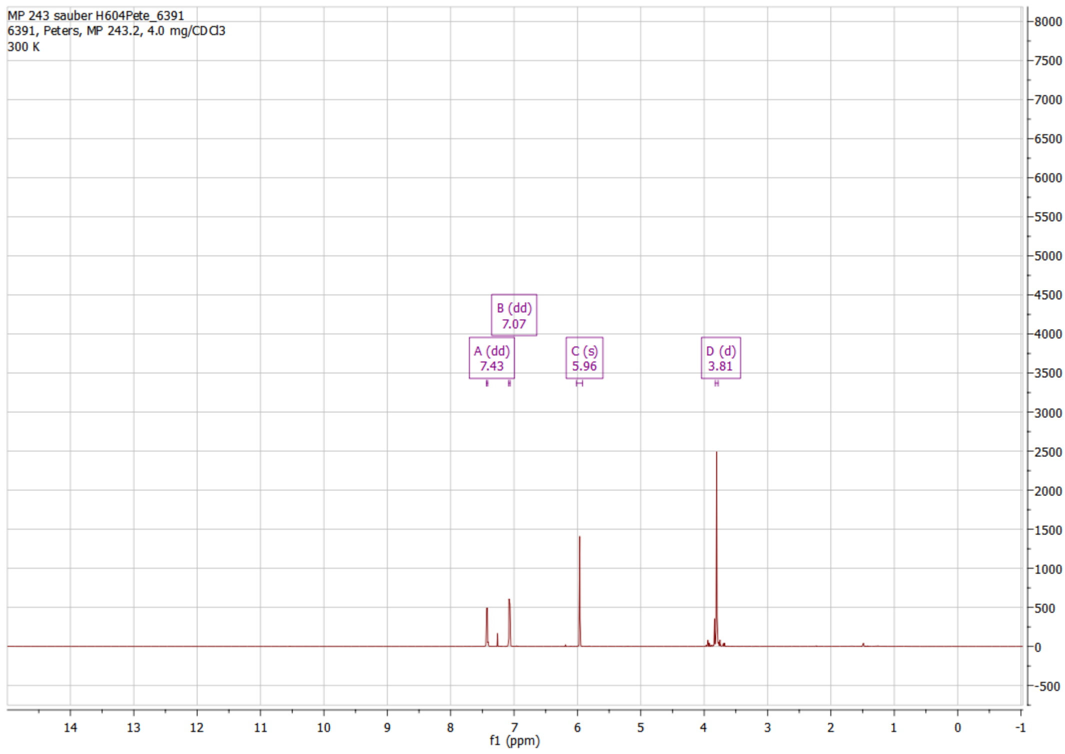

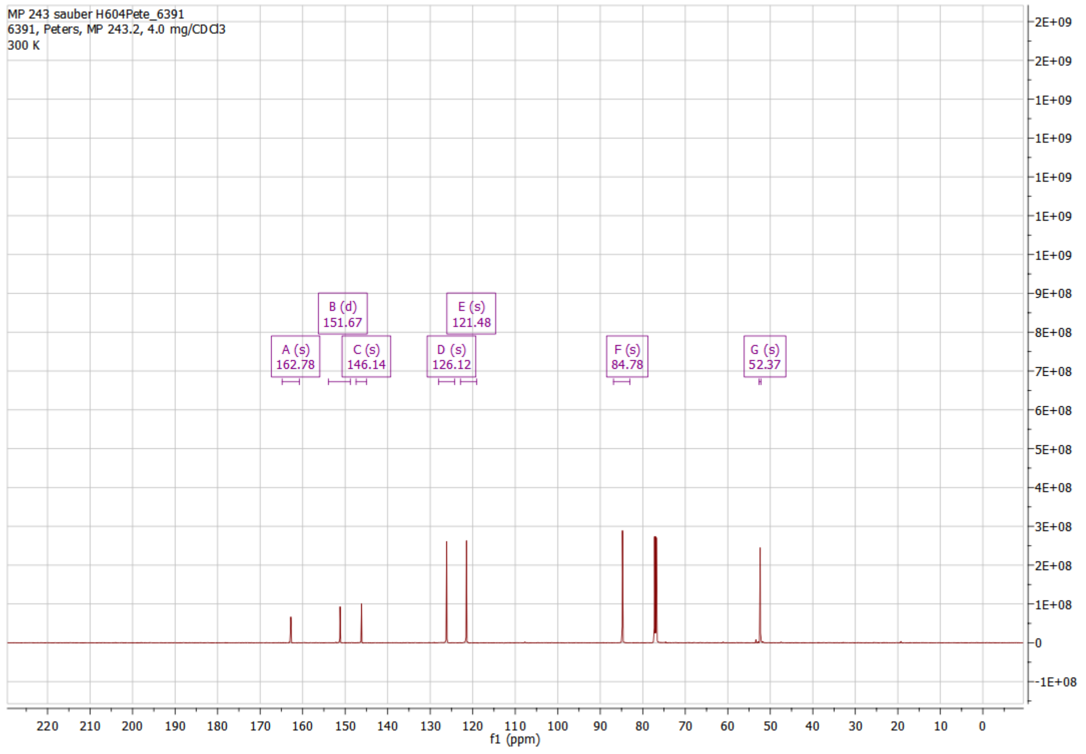

Supplement: File 1 — Analytical equipment and methods, experimental procedures and NMR spectra. [file Beilstein_J_Org_Chem-13-2659-s001.pdf]
